# Effects of blood flow restriction–augmented baduanjin training on quadriceps strength and postural stability in sedentary young men: a randomized controlled trial

**DOI:** 10.1038/s41598-026-53706-x

**Published:** 2026-08-03

**Authors:** Wenbo Ren, Yuanpeng Liao, Zhenhua Jin, Jiahuan Huang, Wenshi Wang

**Affiliations:** 1 Center for Rehabilitation Medicine, Rehabilitation & Sports Medicine Research Institute of Zhejiang Province, Department of Rehabilitation Medicine, Zhejiang Provincial People’s Hospital (Affiliated People’s Hospital), Hangzhou Medical College, Hangzhou, Zhejiang, China; 2 Institute of Sports Medicine and Health, Chengdu Sport University, Chengdu, China

**Keywords:** Baduanjin, Blood Flow Restriction, Exercise Therapy, Muscle Strength, Postural Control, Rehabilitation, Health care, Medical research, Physiology

## Abstract

This randomized controlled trial examined whether augmenting traditional Baduanjin (BDJ) exercise with blood flow restriction (BFR) enhances quadriceps muscle function and postural stability in sedentary young men. A total of 38 participants were randomly assigned to either BDJ or BFR-BDJ training for 8 weeks. Quadriceps peak torque, total work, and single-leg stance stability under eyes-open and eyes-closed conditions were assessed using isokinetic dynamometry and force-platform measures. Compared with BDJ alone, BFR-BDJ produced significantly greater improvements in quadriceps strength and reductions in sway velocity during eyes-open stance. No between-group differences were observed under eyes-closed conditions, suggesting visual dependence in balance enhancement. No adverse events occurred. These findings indicate that integrating BFR with BDJ provides a low-intensity yet effective training strategy to improve lower-limb neuromuscular performance and functional stability in individuals unable or unwilling to engage in high-load exercise.

**Supplementary Information:**

The online version contains supplementary material available at 10.1038/s41598-026-53706-x.

## Introduction

Sedentary behavior is a major global public health issue, associated with declines in physical function, neuromuscular impairments, and elevated risk of chronic diseases^1^. As young adults increasingly adopt inactive lifestyles, accessible and low-intensity strategies that can support musculoskeletal health and functional stability are urgently needed^2^. Complementary mind–body exercises, such as Baduanjin, have gained growing attention as feasible community-based interventions for health promotion^3^.

Baduanjin is a traditional Chinese mind–body practice characterized by gentle movements, regulated breathing, and coordinated postural control. Evidence from clinical and community settings indicates that Baduanjin can improve balance, flexibility, psychological well-being, and cardiometabolic health^4,5^. Due to its low physical demands and minimal equipment requirements, Baduanjin has been widely implemented as a complementary strategy in preventive healthcare and rehabilitation^6^. However, its low-intensity nature may limit its ability to induce significant improvements in muscle strength, particularly in the lower limbs^7^. Lower-limb strength plays a key role in maintaining knee joint stability and controlling center-of-mass displacement during single-leg stance. Enhanced muscle strength may improve joint stiffness regulation and the ability to resist postural perturbations, thereby contributing to improved postural balance^8^.

Blood flow restriction (BFR) training has emerged as a modern, low-load conditioning technique that can safely enhance muscle adaptations with intensities much lower than conventional resistance training^9^. BFR has been incorporated into rehabilitation, community exercise programs, and low-load functional training due to its accessibility and favorable safety profile^10^. BFR is commonly combined with low-load exercises such as walking or cycling; however, these modalities primarily involve repetitive, linear movement patterns and mainly target muscular and cardiovascular adaptations. In contrast, Baduanjin is a mind–body exercise characterized by slow, coordinated, multi-planar movements that integrate breathing control, proprioception, and postural regulation^11^. Therefore, combining BFR with Baduanjin may provide a more comprehensive training stimulus by simultaneously enhancing muscle strength and postural control within a single low-intensity intervention, potentially offering advantages over conventional BFR exercise protocols.

Baduanjin is characterized by slow, controlled movements that involve sustained low-intensity muscle contractions, potentially increasing time under tension. When combined with blood flow restriction, this prolonged contraction pattern may amplify local metabolic stress, promote greater motor unit recruitment, and enhance neuromuscular activation despite the low external load^12^. This theoretical synergy suggests that BFR may compensate for the relatively low mechanical stimulus of Baduanjin. To date, no studies have examined whether combining Baduanjin with BFR can enhance neuromuscular performance in sedentary adults. Such an integrative approach may offer a practical and scalable option for health promotion, particularly for individuals who may be unable or unwilling to engage in high-intensity exercise^13^. Given the well-documented sex-related differences in muscle morphology, hormonal responses, and adaptation to resistance training and blood flow restriction, the present study focused specifically on sedentary young men to reduce physiological variability and enhance internal validity^14^. This approach is consistent with early-stage intervention studies aiming to establish efficacy before extending findings to more diverse populations. Therefore, the aim of this randomized controlled trial was to investigate the effects of BFR-augmented Baduanjin training on quadriceps strength and postural stability in sedentary young men.

## Materials and methods

### Study design

**Fig. 1:**
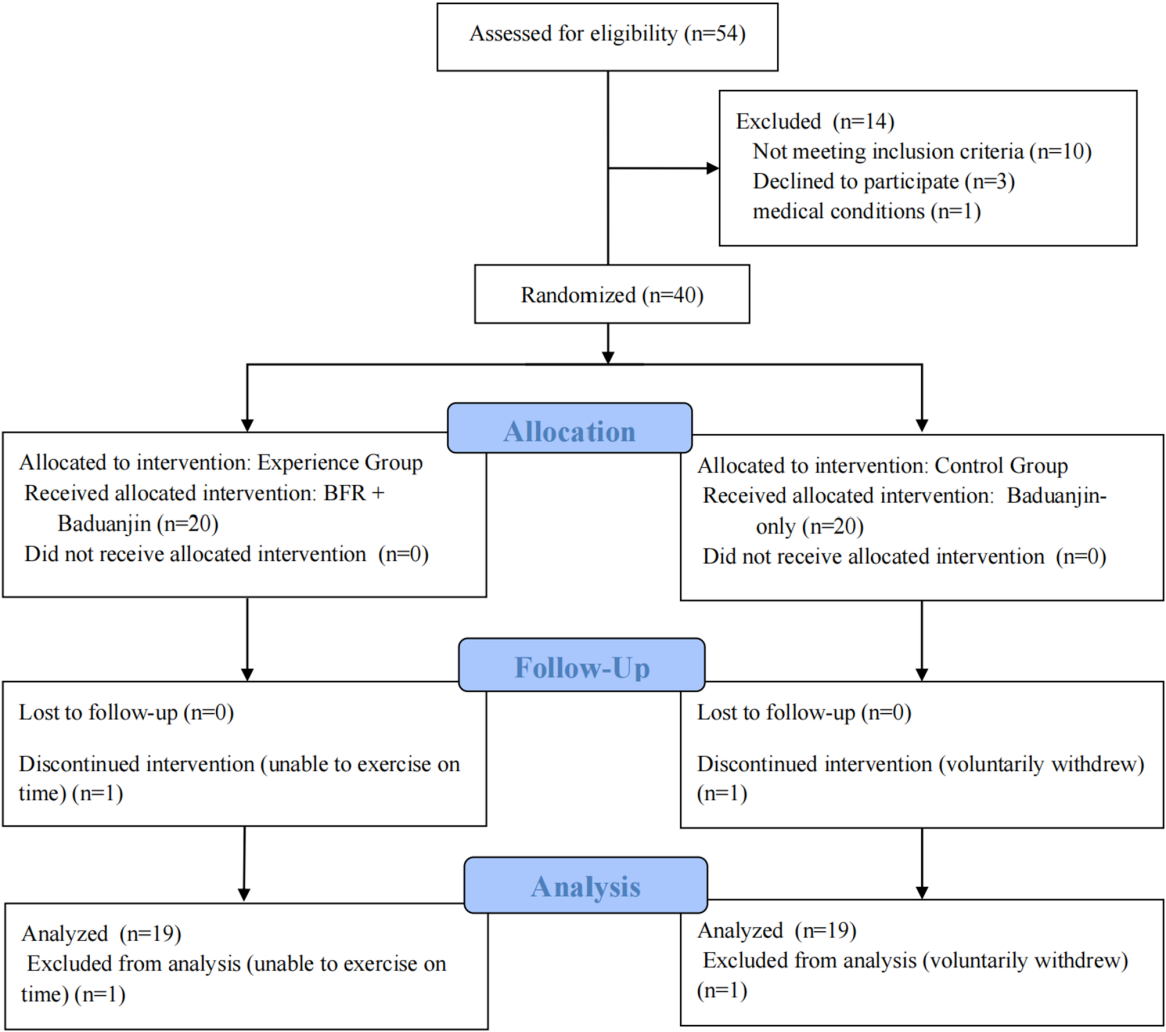
Study flow diagram of the progress through the phases of the experiment.

This study was registered with the China Clinical Trials Registry (ChiCTR2100054120) on December 9, 2021. The procedures were approved by the Ethics Committee of Chengdu Sport University (Approval No. 202165). All procedures involving human participants were performed in accordance with the ethical standards of the institutional research committee and with the 1964 Declaration of Helsinki and its later amendments. Written informed consent was obtained from all participants prior to enrollment. Considering the experimental situation, this research was conducted as a single-blind randomized comparative intervention study, with participants blinded to the specific details of the intervention to prevent bias. Sample size estimation was conducted a priori using G*Power 3.1 based on pilot data (effect size f = 0.35, α = 0.05, power = 0.80), yielding a minimum requirement of 18 participants per group. Each participant was assessed by the same experienced physical therapist before and after the intervention to reduce bias. The data testing and statistical analysts were not involved in the grouping of participants and the intervention (Fig. 1).

### Participants

In this study, only male participants were included to control for potential gender-related differences in muscle strength and response to blood flow restriction. Previous studies have shown that males typically exhibit different physiological responses to resistance training compared to females^15^, and excluding females helped to minimize potential confounding factors in this intervention. Additionally, practical challenges in recruiting female participants, partly influenced by cultural perceptions regarding lower limb muscle aesthetics in this region, also contributed to this decision. The inclusion criteria were as follows:

(1) men aged 18–30 who are in good health; (2) individuals willing to undergo Baduanjin training on time and cooperate in completing various tests. (3) People with low physical activity score according to the International Physical Activity Questionnaire-Short Form (IPAQ-SF).

Exclusion criteria were as follows: (1) individuals who regularly practiced Baduanjin or other Chinese traditional health-Qigong exercises for a long time; (2) individuals in a sports organization, such as martial arts, aerobics, dancing, or Taekwondo; (3) individuals with a recent history of surgery or a significant injury to the locomotor system; (4) individuals suffering from neurological diseases and severe anxiety or depression; (5) individuals with cardiovascular disease and venous thrombosis risk; (6) individuals with a significant abnormal lower-limb alignment in the lower extremities; (7) individuals who are unable to withstand pressure intensity and complete the intervention within the trial’s time limit.

A total of 40 participants completed the baseline assessment and were randomly allocated to either the BFR + BDJ group or BDJ group. Two participants (one per group) withdrew voluntarily during the intervention, leaving 38 for final analysis. There were no significant baseline differences between groups in demographic or anthropometric characteristics (Table 1).

**Table 1:** Participant characteristics (*N* = 38).

Group	*N*	Age	Height	Weight	BMI
		(years)	(cm)	(kg)	(kg/m^2^)
BFR + BDJ	19	27.9 ± 1.8	169.4 ± 7.1	74.13 ± 9.44	21.14 ± 1.90
BDJ	19	28.6 ± 1.9	168.2 ± 6.9	75.08 ± 9.87	20.98 ± 1.85

Note: BMI: body mass index.

## Design and procedures

### Randomization & blinding

The random number and grouping results were generated using the random distribution algorithm in SPSS 26.0 (version 26.0, IBM, Armonk, NY). To meet the experimental standards, participants were randomly assigned to either the BFR + BDJ group (Baduanjin combined with BFR) or the BDJ group in a 1:1 ratio. The randomization envelopes were prepared and preserved by researchers who were not involved in the experimental intervention or the assessment of results. The sealed envelopes were opened in the order that participants completed their first test, and the group assignments were revealed prior to the start of the experiment.

As for blinding, due to the nature of the intervention involving the application of blood flow restriction to the lower limbs, it was not feasible to blind the participants regarding whether or not they received this additional pressure. Therefore, a single-blind design was employed. While the participants were aware of whether they were receiving BFR or not, blinding was applied to the individuals involved in data collection and statistical analysis. This ensured that the testing and analysis of the results were conducted without influence from knowledge of which group the participants had been assigned to, thus minimizing potential bias in the results.

### Measures

Baduanjin training followed the Health Qigong Baduanjin Standard issued by the General Administration of Sport of China (2003)^16^. This system includes the following sections: Sect. 1, elevate both hands to the sky; Sect. 2, draw a bow on both sides; Sect. 3, raise a single arm each time; Sect. 4, look back; Sect. 5, sway the head and shake the tail; Sect. 6, touch toes by hands with flexion of the hip and extension of the knee joint; Sect. 7, clench fists; and Sect. 8, bounce on the toes^17^. The eight sequential movements were instructed and supervised by certified teachers to ensure consistency and accuracy. Participants in both groups trained four sessions per week for 8 consecutive weeks, each lasting approximately 40 min (5-minute warm-up, 30-minute main session, 5-minute cool-down). Blood flow restriction was administered bilaterally at the most proximal portion of the thighs using 5-cm pneumatic cuffs (B-Strong, USA). Cuff pressure was individualized based on thigh circumference, with inflation thresholds set as follows: ≤50 cm, 200 mmHg; 51–55 cm, 250 mmHg; 56–59 cm, 300 mmHg; and ≥ 60 cm, 350 mmHg^18^. The pressure was maintained throughout the 30-minute Baduanjin session and immediately released afterward. Participants were monitored throughout the intervention for any discomfort, numbness, or excessive pain. Participants were instructed to maintain their usual diet and lifestyle habits during the study. To minimize confounding, they completed a standardized weekly log of physical activities and dietary patterns, which was reviewed biweekly by the research team to ensure consistency in habitual behavior.

## Outcome assessments

### Isokinetic muscle strength of the quadriceps

An isokinetic dynamometer was used to measure the isokinetic muscle strength of the quadriceps in both legs (CON-TREX MJ; CMV AG, Zurich, Switzerland). Each test was conducted by the same skilled physiotherapist. The test methods were as follows:

(1) The height, weight, birth date, and other basic details of each participant were recorded.

(2) Through explanation and demonstration, the participants were informed of the requirements and precautions to be taken during the test, and they were asked to complete each test to the best of their ability.

(3) The participants performed a 5-minute bike warm-up before the test to reduce muscle stickiness.

(4) The proximal end of the tested limb and the participant’s trunk were fixed, and each participant was instructed to hold the handles on both sides with both hands without weight bearing on both lower limbs.

(5) Participants were seated with the hip positioned at 105° of flexion and tested in concentric mode at an angular velocity of 60°/s. Each assessment included five consecutive knee flexion and extension cycles to measure peak torque and total work^19^.

(6) The dominant side of the quadriceps was tested first, followed by the opposite side. The participants were allowed to take 2-minute breaks between tests.

(7) Each quadriceps was tested three times on average to reduce errors.

Peak torque was calculated as the maximum force generated by the quadriceps during each test of the isokinetic dynamometry. The peak torque value represents the highest force achieved during the concentric contraction phase of the quadriceps muscle at the specified angular velocity of 60°/s. The highest value from the three trials performed on each leg was recorded and used for statistical analysis. The total work value represents the cumulative effort exerted by the quadriceps during the entire isokinetic exercise session. This measure provides a more comprehensive understanding of muscle endurance and performance^20^. Total work was automatically calculated by the system software as the integrated torque over the range of motion for all repetitions, providing a cumulative measure of mechanical output. Both limbs were assessed in a randomized order, starting from the dominant side, which was identified as the preferred kicking leg.

### Unilateral stance stability

Unilateral stance stability (eyes-open and eyes-closed) was evaluated using the sway velocity measured by the Smart Balance Master (Neurocom, USA). The device consists of a computer processing system, a force-measuring platform, and a force plate. It samples at a frequency of 100 Hz, with one sensor in the horizontal orientation and four sensors in the vertical orientation, to calculate and convert changes in various parameters during the testing process into computer data. Each test was conducted by the same skilled physiotherapist. The test methods were as follows:

(1) The height, weight, age, gender, and other data of the participants were recorded, and the test process and precautions were demonstrated and explained to the participants.

(2) The participants were allowed to stand barefoot on the force plate, facing the front screen and keeping the outside of the heel in the correct position during the test.

(3) The participants were asked to wear a standard fall-prevention harness throughout the test and adjust for suitable tightness.

(4) The dominant lower limbs were tested first, and each test was repeated three times to take the average value (with a 30-second interval between each test).

### Statistical analysis

All statistical analyses were performed using SPSS version 26.0 (IBM, Armonk, NY). All data are presented as mean ± standard deviation (SD). The Shapiro–Wilk test was used to assess the normality of data distribution, and Levene’s test was used to evaluate homogeneity of variance.

To examine the effects of the interventions over time, a two-way repeated-measures analysis of variance (ANOVA) [group (BFR + BDJ vs. BDJ) × time (pre vs. post)] was conducted for each outcome variable. Group × Time interaction effects were assessed separately for the left and right limbs of each parameter. When a significant interaction was detected, post hoc pairwise comparisons were conducted using Bonferroni corrections.

The level of statistical significance was set at *p* < 0.05. Effect sizes were reported using partial eta-squared (η²) values.

## Results

### Peak torque of quadriceps

Significant Group × Time interactions were found for both limbs (Left: F (1,36) = 4.25, *p* = 0.044, ηp² = 0.11; Right: F (1,36) = 4.50, *p* = 0.041, ηp² = 0.12). Post hoc comparisons indicated that the BFR + BDJ group achieved significantly greater increases in peak torque compared with BDJ group (Left: ΔBFR + BDJ=19.42 N·m vs. ΔBDJ = 6.93 N·m; Right: ΔBFR + BDJ=21.71 N·m vs. ΔBDJ = 7.89 N·m). These results confirm that the addition of BFR substantially augmented strength gains beyond those achieved by traditional Baduanjin training (Table 2).

**Table 2:** Changes in peak torque of quadriceps before and after intervention.

Group	Left (*N* · m)		Right (*N* · m)	
	Pre-intervention	Post-intervention	Pre-intervention	Post-intervention
BFR + BDJ	133.24 ± 32.73	152.66 ± 26.72*#	132.03 ± 33.35	153.74 ± 21.50**#
BDJ	128.41 ± 30.18	135.34 ± 28.35	130.78 ± 30.69	138.67 ± 22.69

Note: * indicates *p* < 0.05, ** indicates *p* < 0.01 for within-group comparison (pre- vs. post-intervention); # indicates significant Group × Time interaction (mixed ANOVA: Left F (1,36) = 4.25, *p* = 0.044; Right F (1,36) = 4.50, *p* = 0.041).

### Total work of quadriceps

A similar pattern was observed for total work, with significant Group × Time interactions (Left: F (1,36) = 4.30, *p* = 0.043, ηp² = 0.11; Right: F (1,36) = 4.25, *p* = 0.045, ηp² = 0.10). The BFR + BDJ group exhibited markedly higher increases in total work (Left: + 126.35 N·m; Right: + 134.10 N·m) compared with the BDJ group (Left: + 20.84 N·m; Right: + 30.97 N·m), confirming the synergistic benefit of blood flow restriction (Table 3).

**Table 3:** Changes in total work of quadriceps before and after intervention.

Group	Left (*N* · m)		Right (*N* · m)	
	Pre-intervention	Post-intervention	Pre-intervention	Post-intervention
BFR + BDJ	608.17 ± 156.15	734.52 ± 153.83*#	611.16 ± 155.32	745.26 ± 157.07*#
BDJ	609.17 ± 152.88	630.01 ± 149.40	611.91 ± 156.20	642.88 ± 149.26

Note: * indicates *p* < 0.05 for within-group comparison; # indicates significant Group × Time interaction (mixed ANOVA: Left F (1,36) = 4.30, *p* = 0.043; Right F (1,36) = 4.25, *p* = 0.045).

### Unilateral stance stability (Eyes-open)

Significant interaction effects were detected for sway velocity with eyes-open (Left: F (1,36) = 4.11, *p* = 0.046; Right: F (1,36) = 4.30, *p* = 0.043). The BFR + BDJ group achieved greater reductions in sway velocity (Left: −0.47 deg/s vs. −0.26 deg/s; Right: −0.58 deg/s vs. −0.28 deg/s) compared with BDJ group, indicating superior postural stability (Table 4).

**Table 4:** Changes in sway velocity of unilateral stance (eyes-open) before and after intervention.

Group	Left(deg/s)		Right(deg/s)	
	Pre-intervention	Post-intervention	Pre-intervention	Post-intervention
BFR + BDJ	1.51 ± 0.46	1.04 ± 0.32**#	1.57 ± 0.56	0.99 ± 0.27**#
BDJ	1.53 ± 0.56	1.27 ± 0.34*	1.48 ± 0.60	1.20 ± 0.34*

Note: * indicates *p* < 0.05 for within-group comparison; ** indicates *p* < 0.01 for within-group comparison; # indicates significant Group × Time interaction (mixed ANOVA: Left F (1,36) = 4.11, *p* = 0.046; Right F (1,36) = 4.30, *p* = 0.043).

### Unilateral stance stability (Eyes-closed)

No significant Group × Time interactions were observed under eyes-closed conditions (Left: F (1,36) = 0.03, *p* = 0.86; Right: F (1,36) = 0.001, *p* = 0.97), suggesting that improvements in non-visual balance control were comparable between groups (Table 5).

**Table 5:** Changes in sway velocity of unilateral stance (eyes-closed) before and after intervention.

Group	Left(deg/s)		Right(deg/s)	
	Pre-intervention	Post-intervention	Pre-intervention	Post-intervention
BFR + BDJ	2.25 ± 0.75	1.86 ± 0.60*	2.35 ± 0.82	1.87 ± 0.62*
BDJ	2.26 ± 0.71	1.81 ± 0.66*	2.31 ± 0.73	1.87 ± 0.65*

Note: * indicates *p* < 0.05 for within-group comparison. No significant Group × Time interaction (Left F (1,36) = 0.03, *p* = 0.86; Right F (1,36) = 0.001, *p* = 0.97).

## Discussion

This randomized controlled trial examined whether augmenting Baduanjin training with blood flow restriction (BFR) could enhance neuromuscular adaptations in sedentary young men. The main finding was that the combined intervention produced greater improvements in quadriceps strength and postural stability (eyes-open condition) compared with Baduanjin alone. These results suggest that BFR may help address the recognized limitation of traditional Baduanjin—its insufficient mechanical load for improving lower-limb strength—while preserving its well-documented benefits for balance and coordinated movement.

### Effects on quadriceps strength

Baduanjin augmented with BFR led to significantly greater gains in quadriceps peak torque and total work than Baduanjin alone. This aligns with previous research indicating that BFR can enhance neuromuscular adaptation under low-load conditions exercises^21–24^. Traditional Baduanjin emphasizes gentle, rhythmic motions and mind–body coordination, which are effective for flexibility and postural control but typically do not generate sufficient mechanical stress to stimulate substantial strength improvements^25,26^. The minimal changes observed in the Baduanjin-only group are therefore consistent with earlier studies of other low-intensity exercise modalities.

The addition of BFR may have provided an additional physiological challenge that facilitated strength improvements. Low-load BFR training is thought to induce localized metabolic stress and transient hypoxia, which may contribute to neuromuscular adaptations through mechanisms such as metabolite accumulation, increased motor-unit recruitment, and enhanced muscle fiber activation^27,28^. These processes have been proposed to support muscle protein accretion even when external loads are modest^29^. Although the present study did not assess biochemical markers, the greater gains observed in the BFR + Baduanjin group appear consistent with these previously described responses. Importantly, this approach offers a practical alternative for individuals who may not tolerate high-intensity resistance training^30^, suggesting that the combination of BFR with a culturally accessible exercise such as Baduanjin may broaden the applicability of low-load strength-enhancing interventions.

### Effects on unilateral stance stability

Although outcomes were analyzed based on anatomical left and right limbs to ensure consistency in longitudinal comparisons, group differences and within-subject changes over time remained the primary focus of interpretation. Both groups demonstrated improvements in single-leg stance performance, supporting the established role of Baduanjin in promoting postural control through slow, coordinated, and multi-planar movement^31^. Many Baduanjin sequences involve sustained weight-shifting, trunk control, proprioceptive engagement, and visually guided movement, which are well-suited for enhancing sensory integration and balance^32^. These characteristics likely contributed to the improvements seen in the Baduanjin group.

The combined BFR + Baduanjin intervention, however, yielded a significantly greater reduction in sway velocity in the eyes-open condition. Because dynamic balance relies partly on lower-limb muscle strength, the enhanced quadriceps performance observed in the BFR group may have supported superior stability. BFR training has also been reported to elicit neural or systemic effects beyond the occluded region, which may influence adjacent musculature or motor-control pathways^33,34^. Although the present study did not evaluate hip, trunk, or proximal neuromuscular adaptations, such indirect influences may partly explain the greater improvements observed in the combined group.

However, the differential improvement in “eyes-open” versus “eyes-closed” stability in this study warrants attention. Significant improvement was observed only in the “eyes-open” stability. Balance relies on the intricate integration of visual, vestibular, and proprioceptive systems^35,36^. If the improvement primarily occurred with “eyes-open”, it might imply that while BFR + Baduanjin enhanced the muscular components of balance, its direct impact on non-visual sensory systems (proprioception, vestibular) might be less significant, or functional improvements required visual cues. This observation suggests a potential pattern: the effectiveness of interventions on balance may be modulated by the availability of sensory information, particularly vision, which has implications for designing rehabilitation programs targeting different balance deficits.

### Limitations and future research directions

Considering the absence of a precedent reference in the study of BFR combined with Baduanjin, healthy adults were recruited as participants in this experiment for safety and ethical considerations. While this study provides compelling evidence for the efficacy of BFR + Baduanjin, it is important to acknowledge certain limitations. First, our study design involved a comparison between two active intervention groups (Baduanjin alone vs. Baduanjin + BFR). We acknowledge that the absence of a non-exercise, time-matched control group is a limitation in definitively isolating the absolute effects of BFR. However, our primary objective was to investigate the incremental benefit of BFR when augmenting a low-intensity exercise like Baduanjin. Future research should consider incorporating a non-exercise control group. Second, this study focused primarily on functional outcomes, including muscle strength and postural stability, which are directly relevant to exercise prescription and rehabilitation practice. However, neuromuscular mechanisms such as electromyographic activity, muscle contractile properties, and voluntary activation were not assessed. Future studies integrating electrophysiological and neuromuscular assessments are warranted to provide a more comprehensive understanding of the effects of BFR-augmented Baduanjin training. Third, cuff pressure was prescribed based on thigh circumference rather than individualized arterial occlusion pressure (AOP) or limb occlusion pressure (LOP). While circumference-stratified absolute pressure protocols have been adopted in multiple applied BFR studies and thigh circumference has been identified as the primary predictor of lower-limb occlusion pressure, individualized AOP/LOP-based prescription is acknowledged as the methodological gold standard. Future studies should incorporate individualized pressure assessment to enhance precision and reproducibility across diverse populations.

## Conclusion

This study demonstrated that augmenting Baduanjin exercise with blood flow restriction led to greater improvements in quadriceps strength and eyes-open postural stability compared with Baduanjin alone in sedentary young men. The findings suggest that this low-intensity, hybrid approach may offer a safe and feasible alternative for individuals who cannot tolerate high-load resistance training, supporting its potential application in preventive health and rehabilitation settings. Future studies should explore the applicability of this combined intervention to older adults and clinical populations.

## Electronic Supplementary Material

Below is the link to the electronic supplementary material.

Supplementary Material 1

## Data Availability

The datasets generated during and/or analyzed during the current study are available from the corresponding author on reasonable request.
